# Supplementary figures and images for: Genome-Wide Identification of the StPYL Gene Family and Analysis of the Functional Role of StPYL9a-like in Salt Tolerance in Potato (Solanum tuberosum L.)
Source: Plants (Basel). 2025 Sep 2;14(17):2731. doi: 10.3390/plants14172731 (PMC12430657; doi:10.3390/plants14172731)

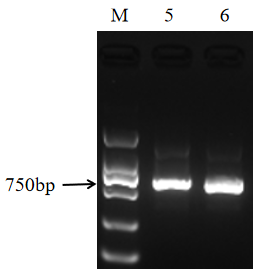

Supplement: Supplementary file 1 [file plants-14-02731-s001.zip › FigureS2.tif]

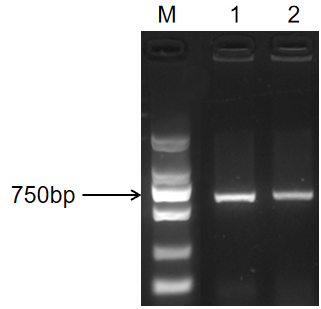

Supplement: Supplementary file 1 [file plants-14-02731-s001.zip › FigureS3.tif]

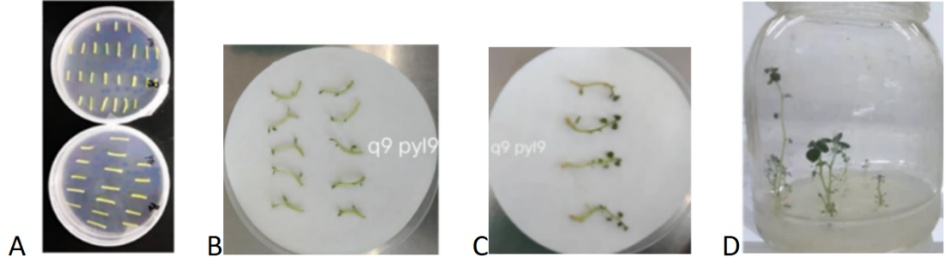

Supplement: Supplementary file 1 [file plants-14-02731-s001.zip › FigureS4.tif]

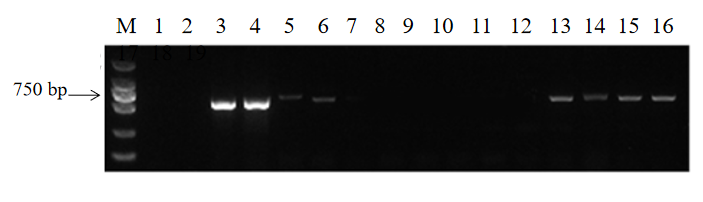

Supplement: Supplementary file 1 [file plants-14-02731-s001.zip › FigureS5.tif]

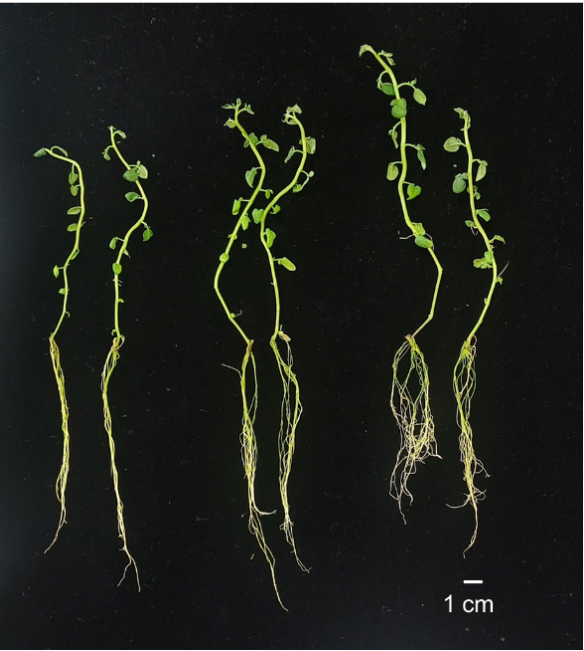

Supplement: Supplementary file 1 [file plants-14-02731-s001.zip › FigureS6.tif]
